# Leiomyosarcoma of the left kidney with renal vein and inferior vena cava tumor thrombus

**DOI:** 10.1016/j.eucr.2025.103221

**Published:** 2025-09-22

**Authors:** Austin Erickson, Aleksander Druck, Andreas Karachristos, Trushar Patel

**Affiliations:** a University of South Florida Health Morsani College of Medicine, 560 Channelside Dr, Tampa, FL 33602, USA; b Department of Urology, University of South Florida, 2 Tampa General Cir, Tampa, FL 33606, USA; c Department of Surgical Oncology, University of South Florida, 2 Tampa General Cir, Tampa, FL 33606, USA

**Keywords:** Renal leiomyosarcoma, Kidney sarcoma, Inferior vena cava tumor thrombus, Radical nephrectomy, Thrombectomy, Rare renal tumors

## Abstract

Primary renal leiomyosarcoma (PRLMS) are rare, aggressive smooth muscle tumors, and vascular involvement with tumor thrombus is especially uncommon. We present a case of a 66-year-old male with hematuria and abdominal pain found to have a 10.5 cm left renal mass with thrombus extending into the renal vein and inferior vena cava (IVC). The patient underwent open radical nephrectomy with IVC thrombectomy. Histopathology confirmed grade 2 leiomyosarcoma with negative surgical margins, The patient recovered uneventfully and remains disease-free five months postoperatively, highlighting the important of complete resection.

## 1 Introduction

Leiomyosarcomas are malignant mesenchymal tumors arising from smooth muscle. Renal leiomyosarcoma is exceedingly rare, representing fewer than 2 % of all primary renal tumors.^1^, ^2^, ^3^, ^4^ Because they often mimic renal cell carcinoma radiographically and clinically, diagnosis is typically confirmed only after surgical excision and histopathologic evaluation.^1^^,^^5^^,^^6^ These tumors are aggressive, with high rates of local recurrence and poor long-term survival.^3^^,^^5^, ^6^, ^7^ They exhibit a female predominance and most frequently occur in the fifth to sixth decade of life.^1^^,^^2^^,^^5^^,^^6^ Clinical presentation is often nonspecific, with hematuria, abdominal pain, or a palpable mass being the most common findings.^6^^,^^7^ Vascular involvement, particularly tumor thrombus within the renal vein or inferior vena cava (IVC), is well recognized in renal cell carcinoma but rarely described in leiomyosarcoma.^8^, ^9^, ^10^, ^11^ Tumor thrombus complicates surgical management and generally worsens prognosis.^4^^,^^9^^,^^10^

We describe a rare case of PRLMS with tumor thrombus extending from the renal vein into the IVC. The patient underwent radical nephrectomy and IVC thrombectomy, achieving margin-negative resection. This case highlights the technical feasibility and clinical importance of complete surgical excision in the setting of vascular involvement.

### 1.1 Case presentation

**Fig. 1:**
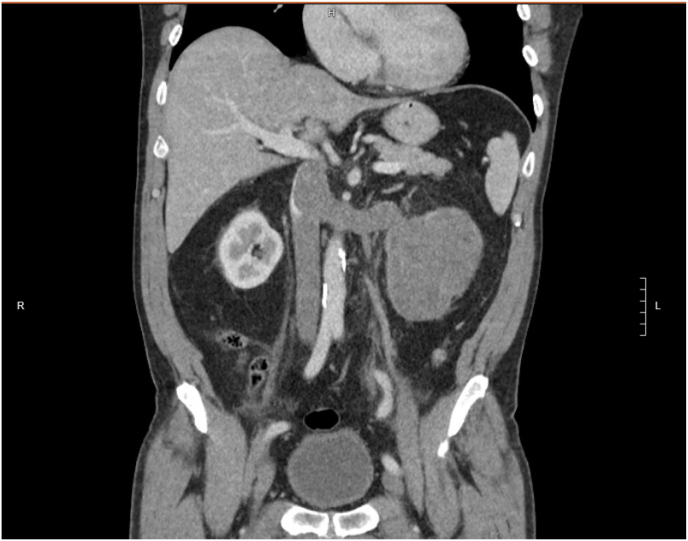
CT scan of abdomen and pelvis showing left renal mass with tumor thrombus in the renal vein and IVC.

**Fig. 2:**
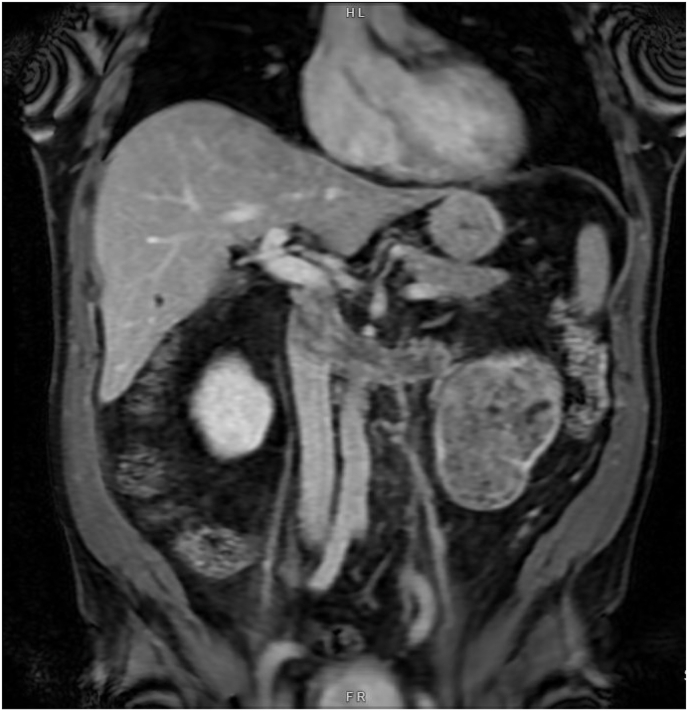
MRI of the abdomen and pelvis showing left renal mass with tumor thrombus occupying the renal vein and IVC.

A 66-year-old obese male presented with a two-week history of gross hematuria and left-sided abdominal pain. He denied fever, weight loss, or systemic symptoms. Laboratory testing was notable only for mild anemia. Preoperative contrast-enhanced CT of the abdomen and pelvis revealed a large, infiltrative mass measuring 10.5 cm arising from the lower pole of the left kidney, with tumor thrombus extending into the renal vein and IVC (Fig. 1). MRI confirmed these findings, demonstrating renal artery encasement and thrombus extending just below the intrahepatic IVC (Fig. 2). No evidence of distant metastasis was seen.

The patient underwent an open radical nephrectomy with IVC thrombectomy via midline abdominal incision. Hilar dissection was technically challenging due tumor size. Estimated blood loss was 1.5 L, and one unit of packed red blood cells was transfused postoperatively. The remainder of the perioperative course was uncomplicated, and he was discharged on postoperative day five.

**Fig. 3:**
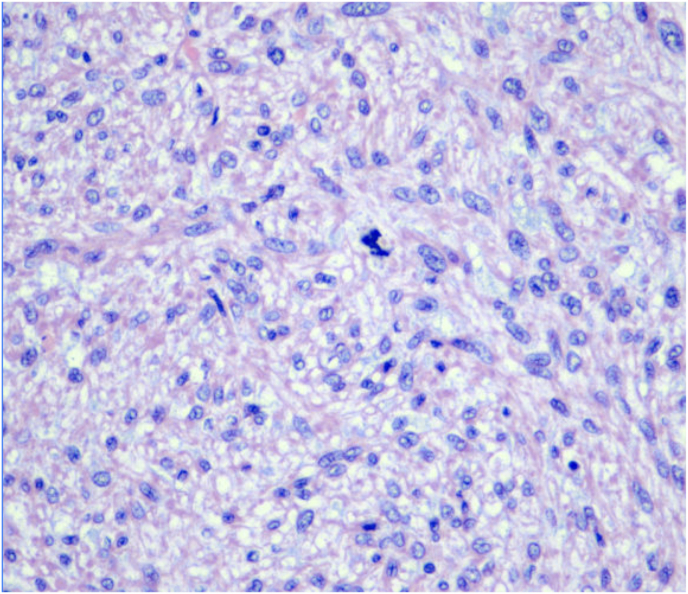
H&E stain revealing a spindle cell malignancy consisting of moderately pleomorphic spindle cells with multifocal areas of necrosis involving approximately 30 % of sampled tumor and an increased number of mitoses (up to 15 in 10 high-power fields) with infrequent abnormal forms.

Gross pathology revealed a centrally located 10.5 cm mass involving the inferior pole with extension into the mid and upper pole. Tumor thrombus was present in the renal vein and its branches but did not invade the IVC wall. Margins were negative. The adrenal gland and para-aortic lymph nodes were free of malignancy. Histopathology confirmed a spindle cell neoplasm with smooth muscle differentiation, consistent with grade 2 leiomyosarcoma (stage IIIB) (Fig. 3). At five months of follow-up, the patient remains clinically well and disease-free, with no radiographic evidence of recurrence or metastasis.

## 2 Discussion

Primary renal leiomyosarcoma is a rare and highly aggressive tumor that can arise from the renal capsule, pelvis, or vasculature.^1^^,^^2^^,^^4^ Because its clinical presentation and imaging often overlap with renal cell carcinoma, definitive diagnosis requires histopathology.^1^^,^^5^^,^^6^ Tumors are usually large at presentation, and many are diagnosed at an advanced stage.^2^^,^^6^^,^^7^

Tumor thrombus in the renal vein or IVC is a hallmark of advanced renal cell carcinoma but has been reported only rarely in renal leiomyosarcoma.^8^^,^^10^^,^^11^ When present, it increases the complexity of surgery and is associated with decreased survival.^4^^,^^9^^,^^10^ In this case, the thrombus extended into the IVC without direct caval wall invasion, allowing negative-margin thrombectomy without vascular reconstruction. This distinction between intraluminal thrombus and direct wall invasion is clinically important, as complete removal without caval resection can facilitate margin clearance and may improve short-term outcomes.^10^^,^^12^^,^^13^

Histopathology confirmed spindle cell morphology with smooth muscle differentiation, consistent with leiomyosarcoma.^1^^,^^2^^,^^6^ Prognosis is generally poor, with survival closely tied to tumor size, grade, and the ability to achieve complete surgical excision.^3^, ^4^, ^5^ Five-year survival rates remain low, highlighting the need for aggressive treatment and close surveillance.^3^^,^^5^ Surgery is the cornerstone of therapy, as combination of chemotherapy and radiotherapy have limited impact on long-term outcomes in localized disease.^3^^,^^5^^,^^6^ Radical resection with thrombectomy has been associated with better survival compared to incomplete excision, even when the IVC is involved.^12^, ^13^, ^14^ The disease-free status of this patient at five months highlights the importance of margin-negative resection, although recurrence remains a concern and long-term vigilance is necessary.^3^^,^^5^

This case also underscores the importance of distinguishing between a renal leiomyosarcoma with contiguous vascular extension and a primary IVC leiomyosarcoma secondarily involving the kidney. The dominant renal mass with continuous thrombus strongly supports a renal origin, which is an important distinction that carries clinical relevance for prognosis and management.^9^^,^^10^^,^^12^

## 3 Conclusion

Primary renal leiomyosarcoma (PRLMS) is a rare tumor with an overwhelmingly poor prognosis. Furthermore, tumor thrombus extension into the renal vein and IVC is incredibly uncommon and complicates surgical management. The combination of these two presentations is a rarely reported finding, and documented cases typically demonstrate a subsequent progression to metastatic disease and mortality. However, this patient has remained disease-free for five months following a margin-negative resection, representing a unique case.

## CRediT authorship contribution statement

**Austin Erickson:** Data curation, Methodology, Writing – original draft. **Aleksander Druck:** Conceptualization, Writing – review & editing. **Andreas Karachristos:** Conceptualization. **Trushar Patel:** Conceptualization, Methodology, Project administration, Supervision, Writing – review & editing.

## Ethics statement

This study was deemed exempt from Institutional Review Board review.

## Funding/Support

None.

## Declaration of competing interest

The Authors have no conflicts of interest to disclose.
